# Supplementary material for: Microbiome homeostasis on rice leaves is regulated by a precursor molecule of lignin biosynthesis
Source: Nat Commun. 2024 Jan 2;15:23. doi: 10.1038/s41467-023-44335-3 (PMC10762202; doi:10.1038/s41467-023-44335-3)
Supplement: Supplementary file 1 — Supplementary Information [file 41467_2023_44335_MOESM1_ESM.pdf]

Microbiome homeostasis on rice leaves is regulated by a precursor molecule of lignin biosynthesis

Su *et al.*

Supplementary Table 1. Assessment of differences in relative abundance of prevalent bacterial phyla and classes of Proteobacteria between *indica* and *japonica* varieties.

**Phylum**	***indica***	***japonica***	***P*-value**
Gammaproteobacteria	78.21±23.19	84.14±19.2	0.027751
Alphaproteobacteria	8.36±10.72	8.21±12.29	0.913264
Betaproteobacteria	3.88±7.31	2.23±3.03	0.027164
Bacteroidetes	3.86±6.42	1.74±2.33	0.00112
Actinobacteria	2.3±2.89	1.53±2.49	0.024157
Firmicutes	1.92±7.53	0.95±2.9	0.203767
Chordata	0.63±1.36	0.59±1.53	0.837923
Deinococcus.Thermus	0.35±0.83	0.14±0.3	0.01048
Cyanobacteria	0.07±0.17	0.08±0.27	0.777662
Deltaproteobacteria	0.06±0.09	0.08±0.17	0.234089

Note: *P*-values were calculated with unpaired one-way ANOVA with Tukey's HSD test. Values are means ± SD of relative abundance (%). The numbers of replicated samples are as follows: *indica* (n=56) and *japonica* (n=36) with three replications for each genotype.

Supplementary Table 2. Assessment of differences in relative abundance between *indica* and *japonica* varieties for the top 20 bacterial orders.

Order	*indica*	*japonica*	*P*-value
Pseudomonadales	56.92±30.36	72.88±28.34	1.73451E-05
Xanthomonadales	1.83±3.89	3.54±9.1	0.032806387
Enterobacterales	19.22±22.85	7.48±13.16	2.11667E-06
Burkholderiales	3.77±7.16	2.13±2.88	0.024217815
Micrococcales	1.49±2.47	0.85±1.79	0.021012023
Deinococcales	0.35±0.83	0.14±0.3	0.010582855
Flavobacteriales	3.36±6.05	1.13±1.77	0.00023697
Propionibacteriales	0.05±0.06	0.03±0.04	0.041850537
Sphingomonadales	5.39±6.55	5.1±8.48	0.74578493
Rhizobiales	1.51±1.99	1.31±1.89	0.399717003
Primates	0.63±1.36	0.59±1.53	0.837999879
Streptomycetales	0.52±0.86	0.46±1.09	0.646357747
Cytophagales	0.12±0.19	0.18±0.27	0.057460113
Caulobacterales	1.26±4.17	1.66±3.68	0.413137727
Clostridiales	0.14±0.35	0.16±0.53	0.667447453
Pasteurellales	0.1±0.24	0.11±0.41	0.709487129
Myxococcales	0.05±0.07	0.07±0.17	0.100847277
Sphingobacteriales	0.36±0.94	0.43±0.83	0.570421219
Bacillales	1.74±7.54	0.76±2.84	0.198027786
Corynebacteriales	0.11±0.14	0.08±0.1	0.071075851
Rhodobacterales	0.08±0.11	0.06±0.08	0.060125817
Rhodospirillales	0.09±0.15	0.06±0.09	0.072925718
Pseudonocardiales	0.05±0.06	0.04±0.07	0.239238378
Oceanospirillales	0.05±0.09	0.05±0.12	0.781636605
Micromonosporales	0.03±0.04	0.02±0.03	0.074266604
Lactobacillales	0.03±0.07	0.02±0.04	0.093776729
Vibrionales	0.03±0.04	0.04±0.07	0.399594135
Neisseriales	0.07±0.19	0.09±0.37	0.720756264
Campylobacterales	0.29±0.58	0.25±0.63	0.612479565

Note: *P*-values were calculated with unpaired one-way ANOVA with Tukey's HSD test. Values are means ± SD of relative abundance (%). The numbers of replicated samples are as follows: *indica* (n=56) and *japonica* (n=36) with three replications for each genotype.

Supplementary Table 3. Assessment of differences in relative abundance between WT, KO and OE rice at bacterial order level.

Order	KO	OE	WT
Pseudomonadales	15.32±6.36c	62.44±15.01a	33.65±6.1b
Xanthomonadales	18.7±12.35a	2.89±4.22b	0.12±0.12b
Flavobacteriales	11.68±10.13a	0.41±0.28b	5.32±4.51ab
Burkholderiales	4.52±3.72a	1.6±1.22ab	0.86±0.45b
Enterobacterales	11.7±7.22a	13.52±17.75a	31.97±22.74a
Sphingomonadales	17.94±11.42a	8.29±11.04a	13.31±10.81a
Rhizobiales	5.33±3.1a	1.27±1.6a	5.01±3.98a
Paenibacillales	0.79±1.47a	5.92±10.59a	4.02±8.89a
Sphingobacteriales	8.18±9.33a	0.32±0.33a	0.45±0.34a
Caulobacterales	2.36±2.36a	0.05±0.05a	2.06±4.2a
Micrococcales	1.18±1.68a	0.08±0.08a	2.01±2.07a
Bacteroidales	0.57±0.33a	1.21±1.78a	0.31±0.14a
Cytophagales	0.75±1a	0.09±0.11a	0.24±0.18a
Lachnospirales	0.1±0.09a	0.32±0.42a	0.06±0.03a
Oscillospirales	0.12±0.07a	0.23±0.23a	0.12±0.07a
SAR11_clade	0.06±0.04a	0.3±0.48a	0.04±0.05a
Bacillales	0±0a	0.16±0.22a	0.09±0.08a

Note: Different letters indicate a significant difference according to the unpaired one-way analysis of variance (ANOVA) with Tukey's HSD test ($P < 0.05$, P-values are listed in Supplementary Data 5). Values are means ± SD of relative abundance (%). The numbers of replicated samples are as follows: WT (n=6), KO (n=6) and OE (n=6). Groups are abbreviated as: wild-type, WT; *OsPAL02*-knocdown, KO; *OsPAL02*-overexpression, OE.

Supplementary Table 4. Analysis of differences in relative abundance of bacterial orders in WT, KO and OE rice lines.

Order	WT	KOline2	KOline3	OEline2	OEline3
Pseudomonadales	32.6±6.34b	18.53±2.5c	16.46±1.87c	61.19±9.21a	71.42±20.4a
Enterobacterales	31.86±30.85a	14.06±2.35a	15.59±2.08a	23.56±24.14a	26.68±20.45a
Sphingomonadales	9.95±11.94ab	9.38±1.93a	8.76±1.78a	0.31±0.17b	0.12±0.04b
Flavobacteriales	8.41±5.92a	17.36±10.71a	10.61±10.98ab	0.21±0.04b	0.34±0.12b
Paenibacillales	7.08±10.94a	0.11±0.02a	0.06±0.03a	12.55±13.65a	0.02±0.06a
Sphingobacteriales	0.31±0.12b	11.82±8.85a	17.55±8.84a	0.07±0.07c	0.13±0.06c
Rhizobiales	3.75±4.65a	4.84±0.34a	4.57±0.3a	0.12±0.03b	0.08±0.05b
Xanthomonadales	0.13±0.17b	16.02±1.07a	16.44±0.71a	0.04±0.03b	0.04±0.04b
Burkholderiales	0.73±0.21b	2.53±0.37a	2.37±0.31a	0.76±0.75b	0.07±0.06c

Note: Different letters indicate a significant difference according to the unpaired one-way analysis of variance (ANOVA) with Tukey's HSD test ($P < 0.05$, *P*-values are listed in Supplementary Data 5). Values are means ± SD of relative abundance (%). The numbers of replicated samples are as follows: WT (n=6), KOline2 (n=6), KOline3 (n=6), OEline2 (n=6) and OEline3 (n=6). Groups are abbreviated as: wild-type, WT; *OsPAL02*-knockdown line 2, KOline2; *OsPAL02*-knockdown line 3, KOline3; *OsPAL02*-overexpression line 2, OEline2; *OsPAL02*-overexpression line 3, OEline3.

Supplementary Table 5. Analysis of differences in alpha diversity indices between WT, KO and OE rice lines.

Alpha diversity	WT	KOline2	KOline3	OEline2	OEline3
observed_species	85.33±20.72ab	136.5±21.26a	125±27.99a	51.5±13.2c	55.67±13.59bc
Shannon	2.36±0.52b	3.09±0.1a	3.13±0.15a	1.88±0.32b	1.86±0.38b
Simpson	0.81±0.14ab	0.91±0.01a	0.92±0.01a	0.69±0.11b	0.72±0.08b
Pielou	0.53±0.09ab	0.63±0.01ab	0.65±0.02a	0.48±0.09b	0.47±0.1b

Note: Different letters indicate a significant difference according to unpaired one-way analysis of variance (ANOVA) with Tukey's HSD test (P < 0.05, *P*-values are listed in Supplementary Data 5). Values are means ± SD of relative abundance (%). The numbers of replicated samples are as follows: WT (n=6), KOline2 (n=6), KOline3 (n=6), OEline2 (n=6) and OEline3 (n=6). Groups are abbreviated as: wild-type, WT; *OsPAL02*-knockdown line 2, KOline2; *OsPAL02*-knockdown line 3, KOline3; *OsPAL02*-overexpression line 2, OEline2; *OsPAL02*-overexpression line 3, OEline3.

Supplementary Table 6. Analysis of 4-HCA concentrations in WT, KO and OE rice lines.

Genotype	4-HCA (mg/g)
WT	2.749±0.083b
KOline1	1.1±0.125c
KOline2	1.049±0.187c
KOline3	1.146±0.065c
OEline1	8.886±2.029a
OEline2	9.218±1.379a
OEline3	9.834±1.613a

Note: Different letters indicate a significant difference according to the unpaired one-way analysis of variance (ANOVA) with Tukey's HSD test ($P < 0.05$, *P*-values are shown in Supplementary Figure 11). Values are means ± SD of relative abundance (%). The numbers of replicated samples are as follows: WT (n=15), KOline1 (n=15), KOline2 (n=15), KOline3 (n=15), OEline1 (n=15), OEline2 (n=15) and OEline3 (n=15). Groups are abbreviated as: wild-type, WT; *OsPAL02*-knockdown line 1, KOline1; *OsPAL02*-knockdown line 2, KOline2; *OsPAL02*-knockdown line 3, KOline3; *OsPAL02*-overexpression line 1, OEline1; *OsPAL02*-overexpression line 2, OEline2; *OsPAL02*-overexpression line 3, OEline3.

Supplementary Table 7. Analysis of 4-HCA concentrations in *indica* and *japonica* plants.

Subgroup	4-HCA (mg/g)
indica	1.57±0.2073
japonica	3.013±0.5723
P-value	2.43e-36

Note: The *P*-value was calculated with unpaired one-way ANOVA with Tukey's HSD test. Values are means ± SD of relative abundance (%). The numbers of replicated samples are as follows: *indica* (n=75) and *japonica* (n=75).

Supplementary Table 8. Assessment of differences in relative abundance between WT and KO+4HCA rice at bacterial order level.

Order	WT	KO+4HCA	*P*-value
Pseudomonadales	51.88±10	49.13±6.65	0.589
Sphingomonadales	5.83±4.81	9.82±8.87	0.362
Paenibacillales	4.68±7.67	1.65±3.27	0.403
Flavobacteriales	3.13±4.19	1.2±1.2	0.322
Enterobacterales	20.3±21.19	26.42±19.84	0.617
Rhizobiales	2.67±3.98	3.06±2.54	0.847
Burkholderiales	2.59±2.48	2.14±2.96	0.779
Bacteroidales	2.3±3.76	0.57±0.42	0.313
Sphingobacteriales	0.94±1.97	1.95±3.11	0.522
Caulobacterales	0.93±2.13	0.47±0.64	0.627
Xanthomonadales	0.71±0.69	1.13±1.34	0.515
Lachnospirales	0.65±1.17	0.13±0.1	0.327
SAR11_clade	0.57±1.08	0.1±0.07	0.337
Micrococcales	0.55±0.72	0.37±0.53	0.63
Oscillospirales	0.46±0.61	0.18±0.09	0.312
Rhodobacterales	0.32±0.61	0.03±0.03	0.304
Cytophagales	0.23±0.24	0.23±0.49	0.985
Bacillales	0.21±0.47	0.35±0.57	0.657
Lactobacillales	0.12±0.11	0.2±0.23	0.451
Exiguobacterales	0.07±0.06	0.58±0.95	0.246

Note: *P*-values were calculated with unpaired one-way ANOVA with Tukey's HSD test. Values are means ± SD of relative abundance (%). The numbers of replicated samples are as follows: WT (n=6), KO (n=6) and OE (n=6). Groups are abbreviated as: wild-type, WT; *OsPAL02*-knockdown rice mutant supplemented with 4-HCA, KO+4-HCA.

Supplementary Table 9. Oligonucleotide primers used in this study.

Primer name	Sequence (5'→3')	Purpose/function
U6-2	GGACGAGGTGAAGCGCATGGCGGCAGCCAAGCCAGCA	Generation of *OsPAL02-KO* construct
U6-1	CTCCGTTTTACCTGTGGAATCG	Generation of *OsPAL02-KO* construct
gRNA-1	CCATGCGCTTCACCTCGTCCGTTTTAGAGCTAGAAAT	Generation of *OsPAL02-KO* construct
gRNA-2	CGGAGGAAAATTCCATCCAC	Generation of *OsPAL02-KO* construct
sgRNA-1	TTCAGAGGTCTCTACCGACTAGTATGGAATCGGCAGCAAAGG	Generation of *OsPAL02-KO* construct
sgRNA-2	AGCGTGGGTCTCG CTCG ACGCGTATCCATCCACTCCAAGCTC	Generation of *OsPAL02-KO* construct
KO-1	GCGGTGTCATCTATGTTACTAG	Identification of *OsPAL02-KO* construct
KO-2	TGCAATAACTTCGTATAGGC	Identification of *OsPAL02-KO* construct
PAL02-1	ACTAGGGTCTCGCACC ATGGCGAGCCAGACCGCCGACG	Generation of *OsPAL02-OE* construct
PAL02-2	ACTAGGGTCTCTACCG TCAGCAGATGGGCAGGGGCTCGCC	Generation of *OsPAL02-OE* construct
OE-1	TTAGCCCTGCCTTCATACGC	Identification of *OsPAL02-OE* construct
OE-2	ATCATCGCAAGACCGGCAAC	Identification of *OsPAL02-OE* construct
KO2-1	TCTCAGCTCTTCTTCCACCACC	Identification of *OsPAL02-KO* plant
KO2-2	GGGCGATGCAGTTGAGGAT	Identification of *OsPAL02-KO* plant
H-1	TGTAGTGTATTGACCGATTCCTTGC	Identification of *OsPAL02-OE* plant
H-2	GTTCGACAG C GTCTCCGACCTGAT	Identification of *OsPAL02-OE* plant
343F	TACGGRAGGCAGCAG	Primer for high throughput sequencing
798R	AGGGTATCTAATCCT	Primer for high throughput sequencing
27F	AGAGTTTGATCCTGGCTCAG	Identification of isolated taxa
1492R	TACGGCTACCTTGTTACGACTT	Identification of isolated taxa

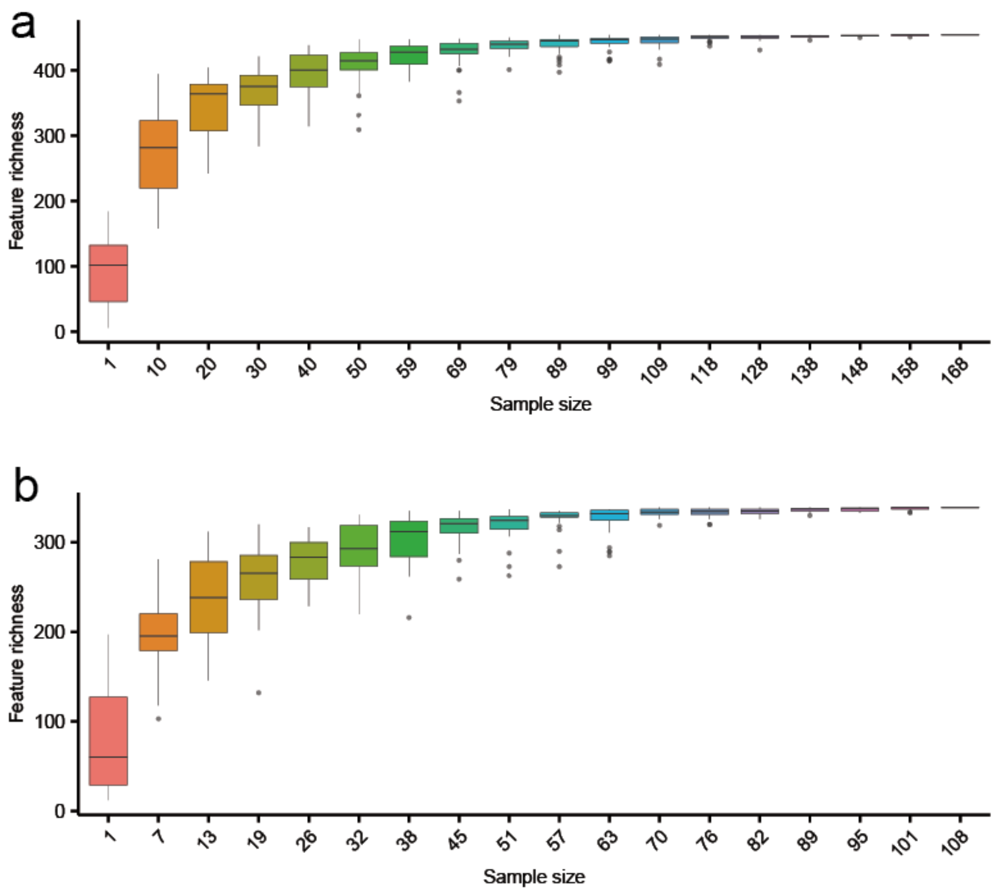

Supplementary Fig. 1. Coverage of phyllosphere microbiomes for *indica* and *japonica* rice varieties. (**a** and **b**) Rarefaction curves of detected bacterial genera reach the saturation stage with increasing numbers of samples, indicating that most leaf bacteria were captured for each rice subspecies. *indica* (**a**) and *japonica* (**b**) varieties are shown separately. The horizontal bars within boxes represent the median. The tops and bottoms of boxes represent 75th and 25th percentiles, respectively. The upper and lower whiskers extend to data no more than 1.5 the interquartile range from the upper edge and lower edge of the box, respectively. The numbers of replicated samples are as follows: *indica* (n=56) and *japonica* (n=36) with three replications for each genotype. Source data are provided as a Source Data file.

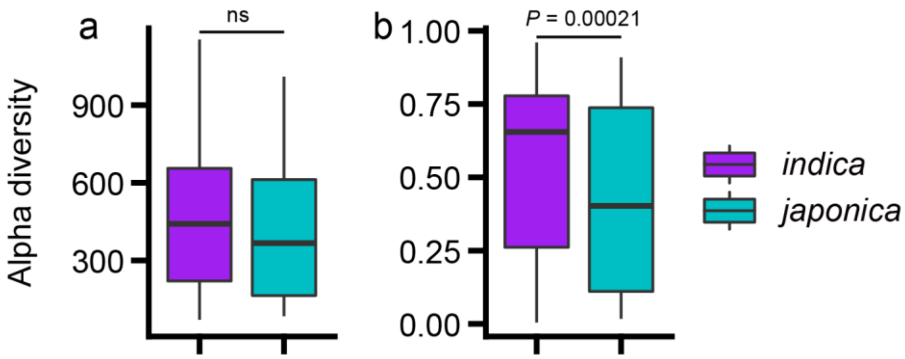

Supplementary Fig. 2. Comparison of within-sample diversity (alpha-diversity) between *indica* and *japonica* plants. (**a** and **b**) richness (**a**) and Simpson index (**b**) of the phyllosphere microbiome of *indica* and *japonica* varieties. The *P*-value was calculated with unpaired one-way ANOVA with Tukey's HSD test. The label 'ns' indicates a not significant difference ($P > 0.05$). In this figure, box plot percentiles are the same as in Supplementary Fig. 1. The numbers of replicated samples are as follows: *indica* (n=56) and *japonica* (n=36) with three replications for each genotype. Source data are provided as a Source Data file.

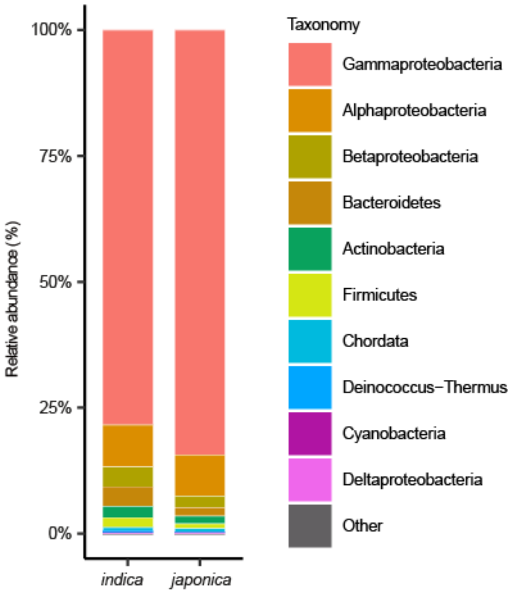

Supplementary Fig. 3. Phylum-level composition of the *indica* and *japonica* phyllosphere microbiomes. The relative abundance of *indica* and *japonica* phyllosphere microbiome members is shown at the phylum level. Proteobacteria are shown at class level. The numbers of replicated samples are as follows: *indica* (n=56) and *japonica* (n=36) with three replications for each genotype. Source data are provided as a Source Data file.

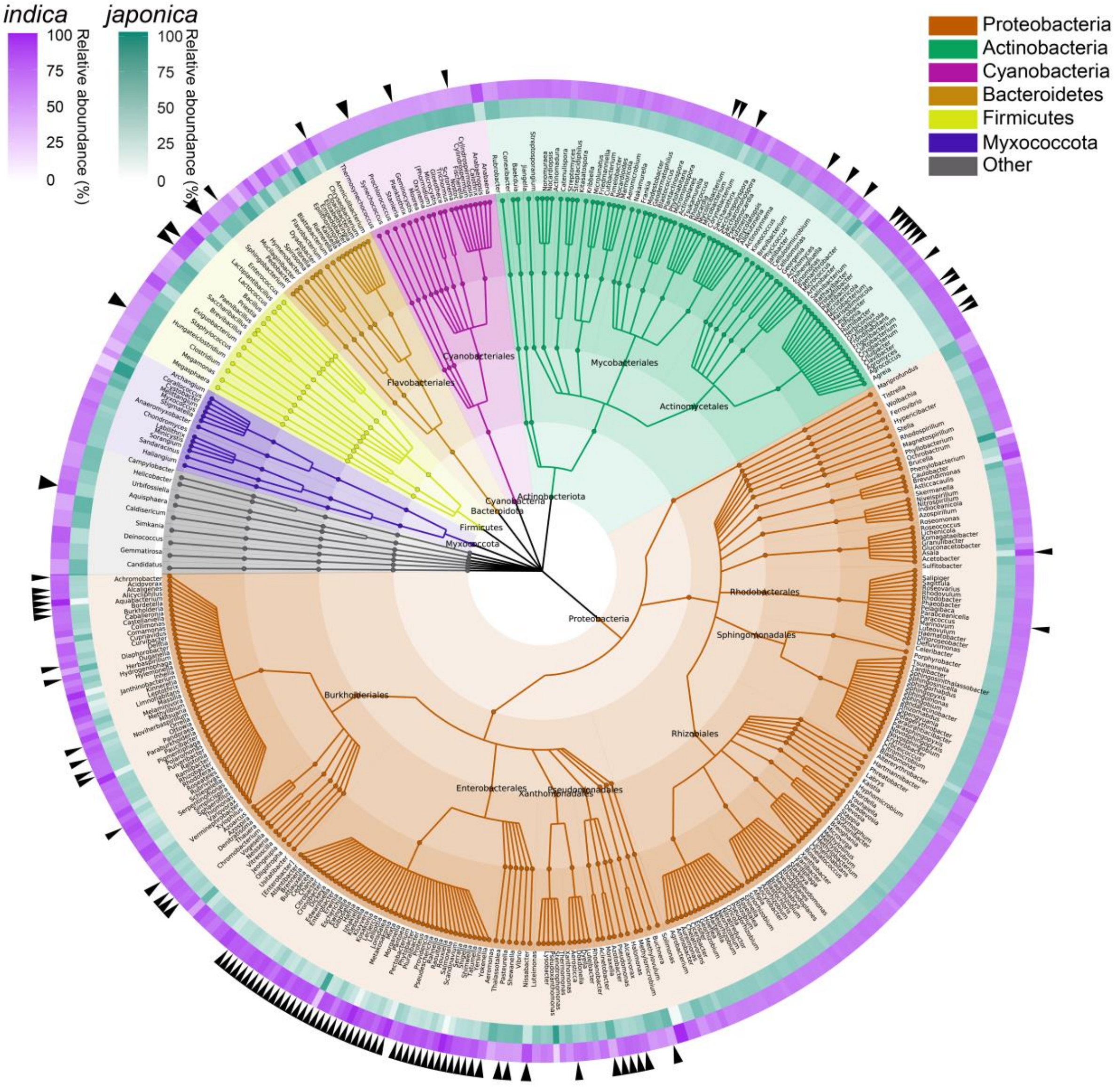

Supplementary Fig. 4. Genus-level composition of the *indica* and *japonica* phyllosphere microbiomes. The inner part represents the phylogeny of all bacterial genera with a relative abundance > 0.001% from *indica* and *japonica* phyllosphere microbiomes. The outer purple and green rings represent their relative abundance in *indica* and *japonica*, respectively. Black triangles indicate significant difference in relative abundance between *indica* and *japonica* (FDR adjusted $P < 0.05$, two-sided Wilcoxon rank sum test, *P*-values are listed in Supplementary Data 2). The numbers of replicated samples are as follows: *indica* (n=56) and *japonica* (n=36) with three replications for each genotype. Source data are provided as a Source Data file.

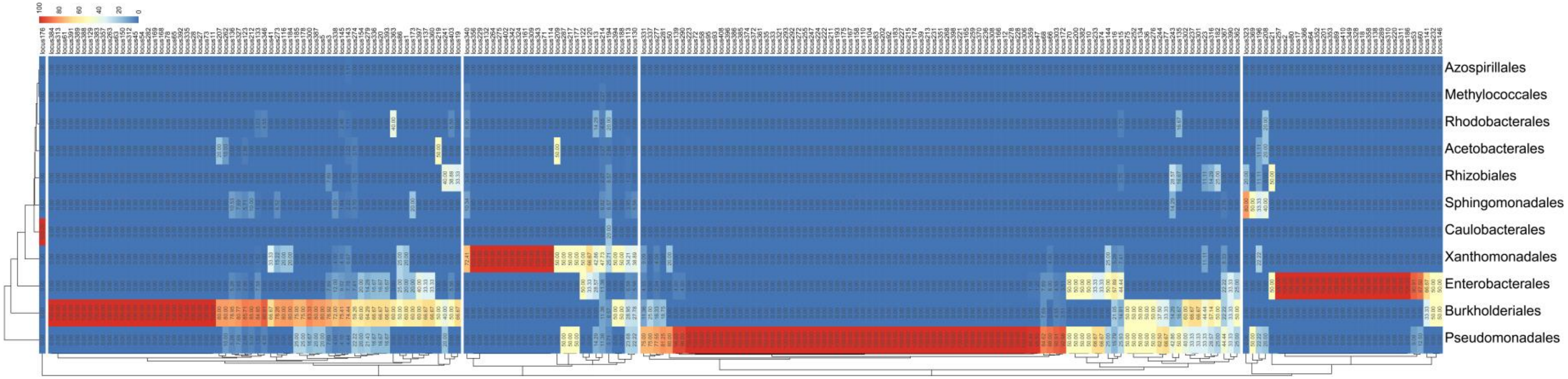

Supplementary Fig. 5. GWAS loci associated with specific bacterial taxa. The heatmap indicates species frequency (summarized at bacterial order level) for each locus. Clusters are marked on the left and bottom using Bray-Curtis distances. All GWAS loci (indicated in the top part of the figure) are shown in Supplementary Data 4. Source data are provided as a Source Data file.

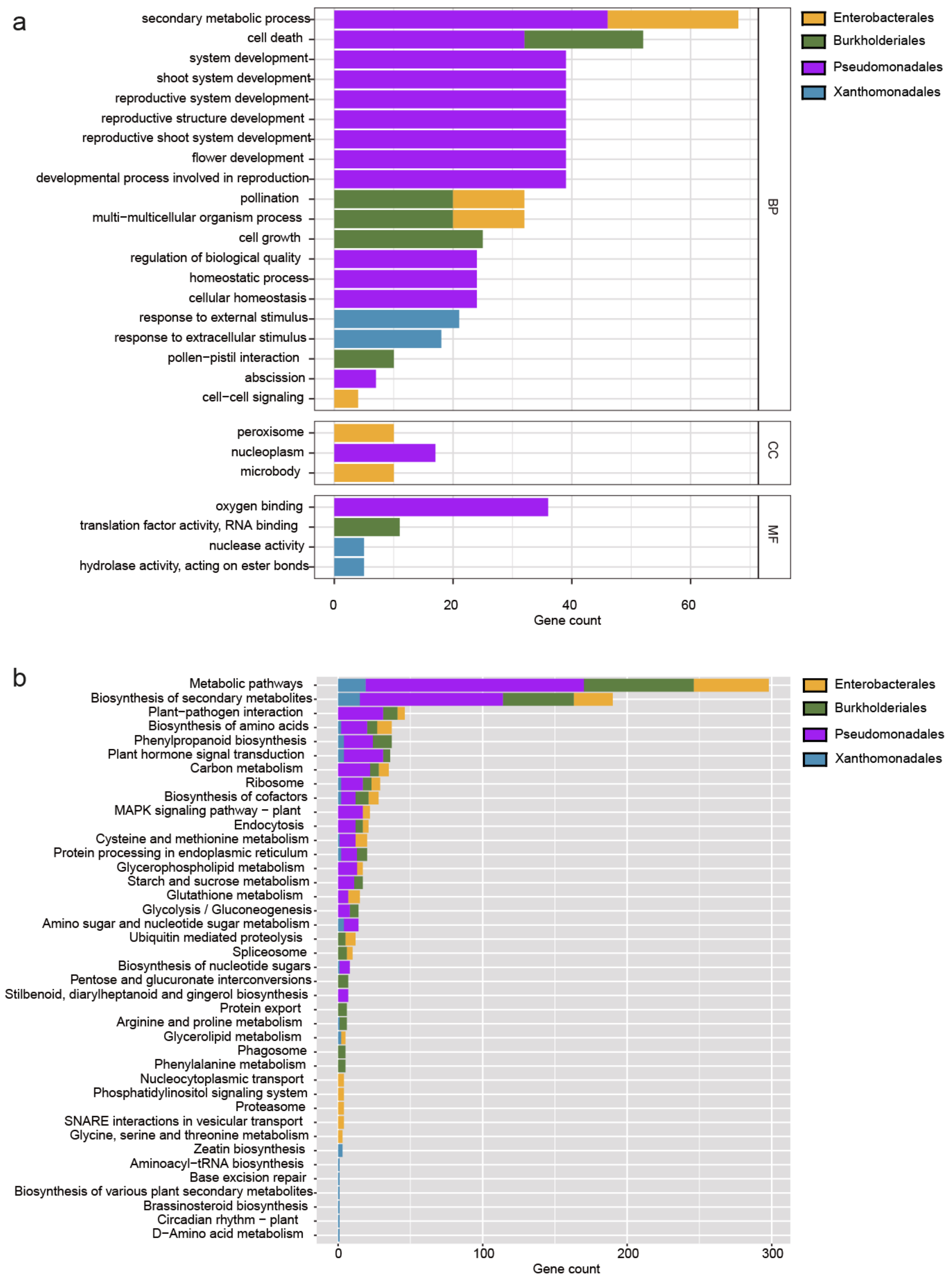

Supplementary Fig. 6. Microbial genes enriched in metabolite pathways. (a) GO pathway enrichment analysis of genes from GWAS locus cluster in Fig. 2a. Gene counts are color coded according to their order-level annotation (right). The bars present significantly enriched gene numbers in each pathway (two-side Fisher's exact test, $P < 0.05$, P-values are listed in Supplementary Data 4). GO terms are abbreviated as: Biological Process, BP; Cellular Component, CC; Molecular Function, MF. **(b)** Genes from GWAS locus cluster in Fig. 2a annotated with KEGG pathways. Gene counts are color coded according to their order-level annotation (right). The bars present numbers of associated genes for each pathway. Source data are provided as a Source Data file.

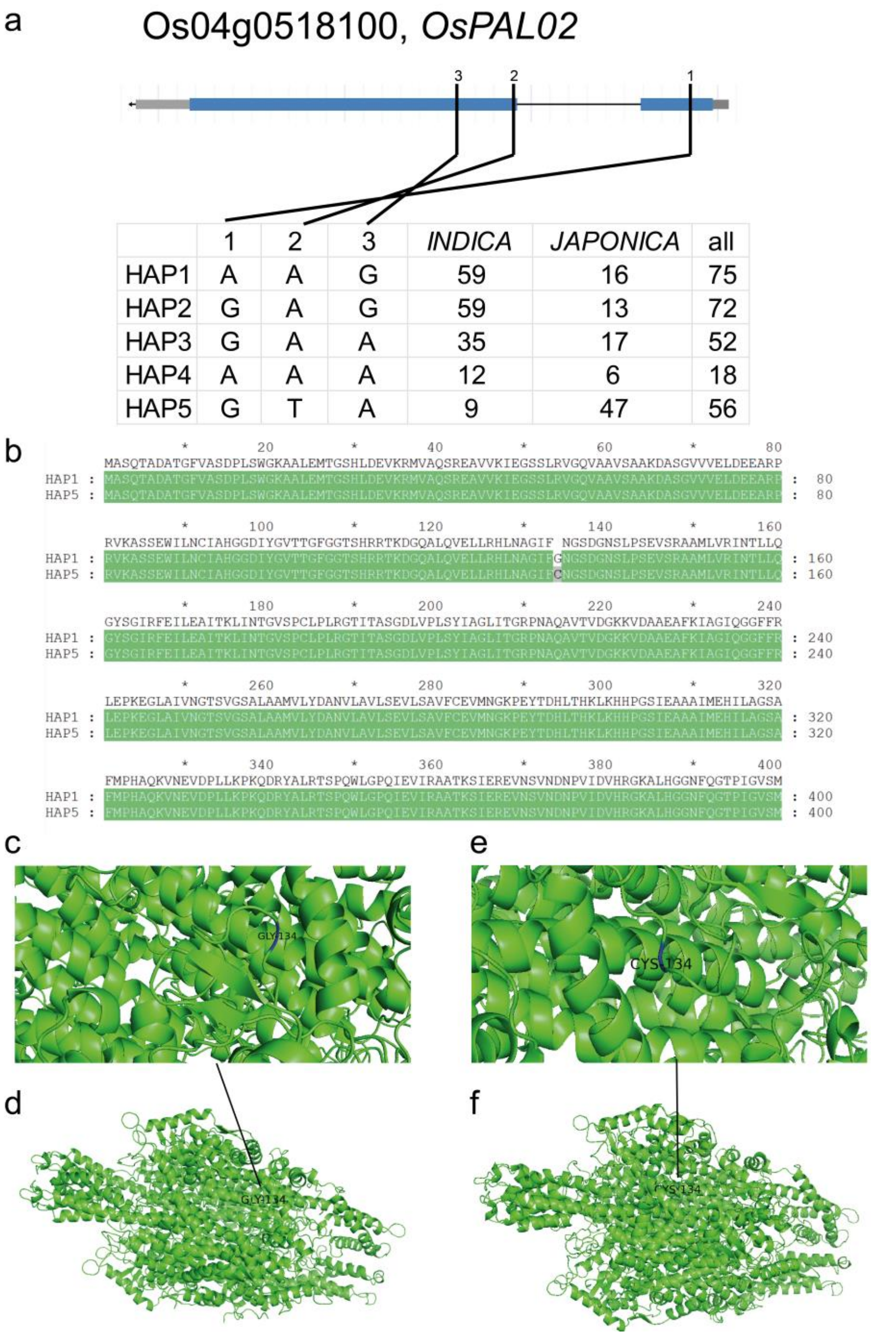

Supplementary Fig. 7. Haplotype analysis of the polymorphic *OsPAL02* CDS region from 295 rice varieties. (**a**) Main haplotype differences between *indica* and *japonica*. (**b**) Alignment of protein sequence for haplotype 1 and haplotype5 using cluster W. Green color indicates sequence similarity of 100%. White and gray color indicates differences in amino acids. Haplotype 1 protein structure (**d**) and differences in amino acids in detail (**c**). Haplotype 5 protein structure (**f**) and differences in amino acids in detail (**e**). Blue color indicates the differences in the structure site. Hap 1, 2, 3, 4, 5 indicates the haplotypes 1, 2, 3, 4, 5. Source data are provided as a Source Data file.

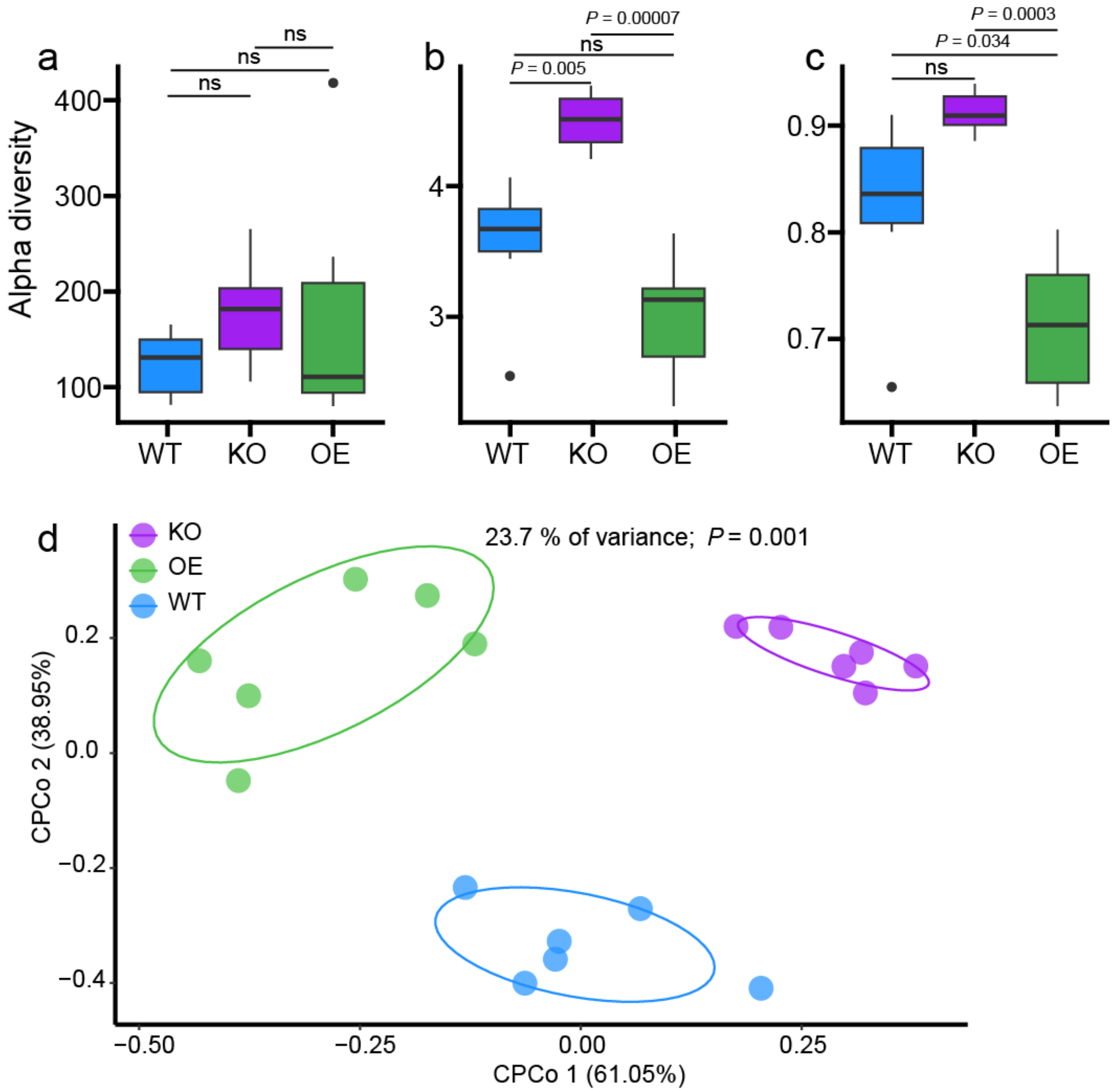

Supplementary Fig. 8. Comparison of within-sample diversity (alpha-diversity) and CPCoA between WT, KO and OE rice phyllosphere microbiomes. Richness (**a**), Shannon index (**b**) and Simpson index (**c**) of the phyllosphere microbiomes of WT, KO and OE rice. (**d**) Constrained PCoA based on Bray-Curtis distances indicates phyllosphere microbiome separation for WT, KO and OE plants (23.7% of total variance was explained by the plant genotype, P = 0.001, P-value was calculated with PERMANOVA). Ellipses cover 68% of the data for each group. Blue, purple and green colors represent WT, KO and OE rice plant samples, respectively. The numbers of replicated samples are as follows: WT (n=6), KO (n=6) and OE (n=6). The P-values were calculated with unpaired one-way ANOVA with Tukey's HSD test. The labels 'ns' indicate a not significant difference (P > 0.05). In this figure, box plot percentiles are the same as in Supplementary Fig. 1. Groups are abbreviated as: wild-type, WT; *OsPAL02*-knockdown line1, KO; *OsPAL02*-overexpression line1, OE. Source data are provided as a Source Data file.

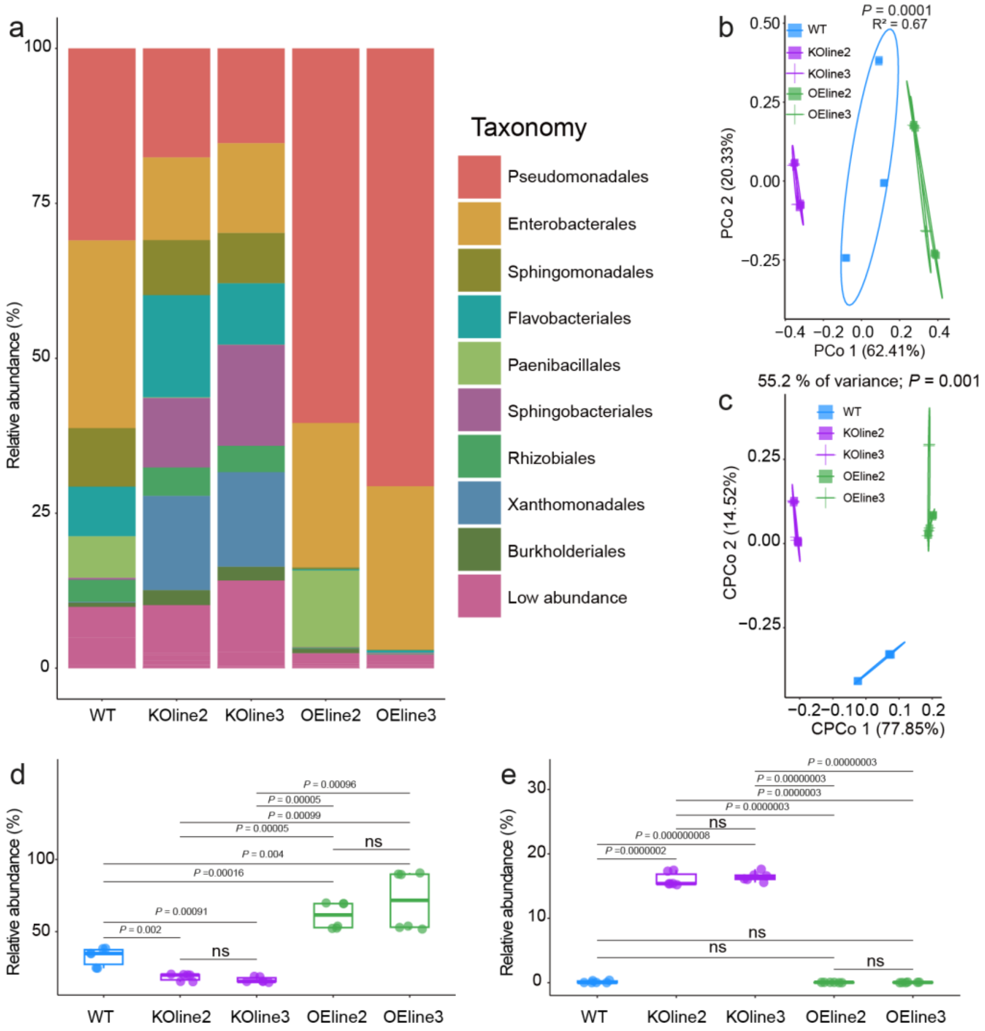

Supplementary Fig. 9. Phyllosphere microbiome of two additional KO and OE lines. (**a**) Bacterial communities at order level in WT (wild-type, ZH11 rice variety), KOline2, KOline3, OEline2 and OEline3 plants. (**b**) Unconstrained PCoA based on Bray-Curtis distances showing bacterial community clustering in WT, KO and OE lines ($P < 0.001$, R^2=0.68, P-value was calculated with PERMANOVA). Ellipses cover 68% of the data for each group. (**c**) Constrained PCoA based on Bray-Curtis distances indicates phyllosphere microbiome separation for WT, KO and OE lines (53.9% of total variance was explained by the plant genotype, $P < 0.001$, P-value was calculated with PERMANOVA). Ellipses cover 68% of the data for each group. (**d** and **e**) Comparison of relative abundances of Pseudomonadales (**d**) and Xanthomonadales (**e**) in WT, KO and OE lines. The numbers of replicated samples in A, B, C, D, E are as follows: WT (n=6), KOline2 (n=6), KOline3 (n=6), OEline2 and OEline3 (n=6). The P-values were calculated with unpaired one-way ANOVA with Tukey's HSD test. The labels 'ns' indicate a not significant difference ($P > 0.05$). In this figure, box plot percentiles are the same as in Supplementary Fig. 1. Groups are abbreviated as: wild-type, WT; *OsPAL02*-knockdown line 2, KOline2; *OsPAL02*-knockdown line 3, KOline3; *OsPAL02*-overexpression line 2, OEline2; *OsPAL02*-overexpression line 3, OEline3. Source data are provided as a Source Data file.

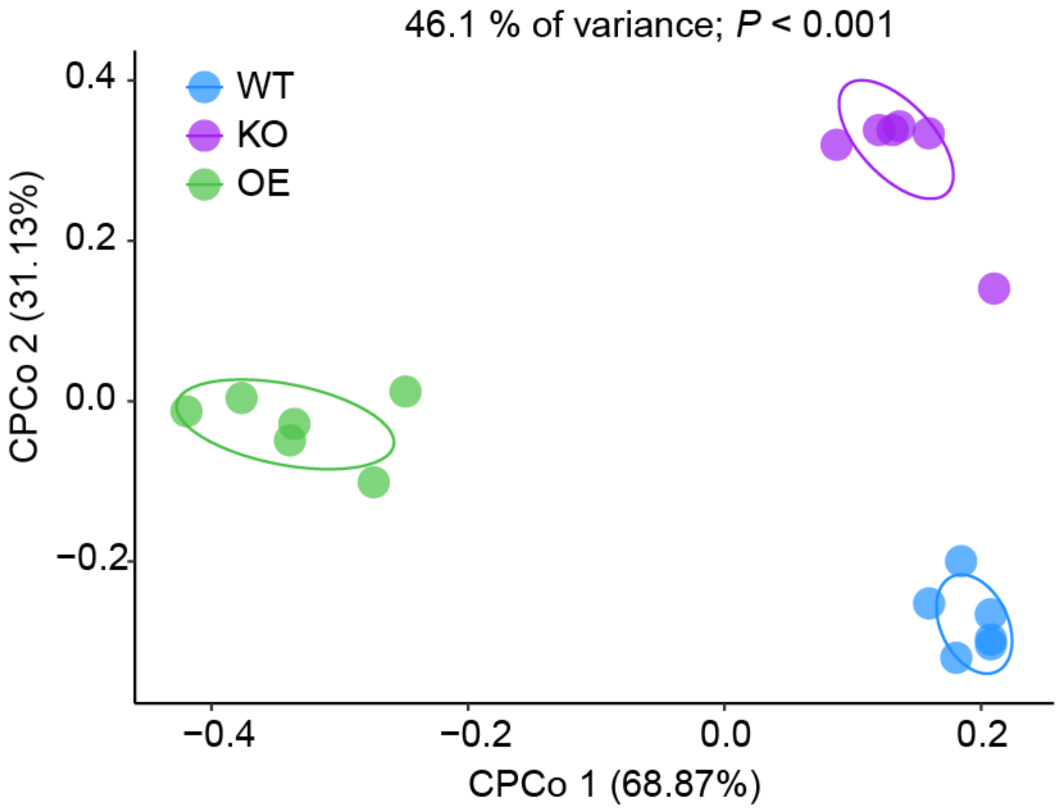

Supplementary Fig. 10. Constrained PCoA of leaf metabolites. The CPCoA based on Bray-Curtis distances shows leaf metabolite separation for WT, KO and OE plants (46.1% of total variance was explained by the plant genotypes, P = 0.0009, P-value was calculated with PERMANOVA). Ellipses cover 68% of the data for each group. Groups are abbreviated as: wild-type, WT; *OsPAL02*-knockdown line 1, KO; *OsPAL02*-overexpression line 1, OE. Source data are provided as a Source Data file.

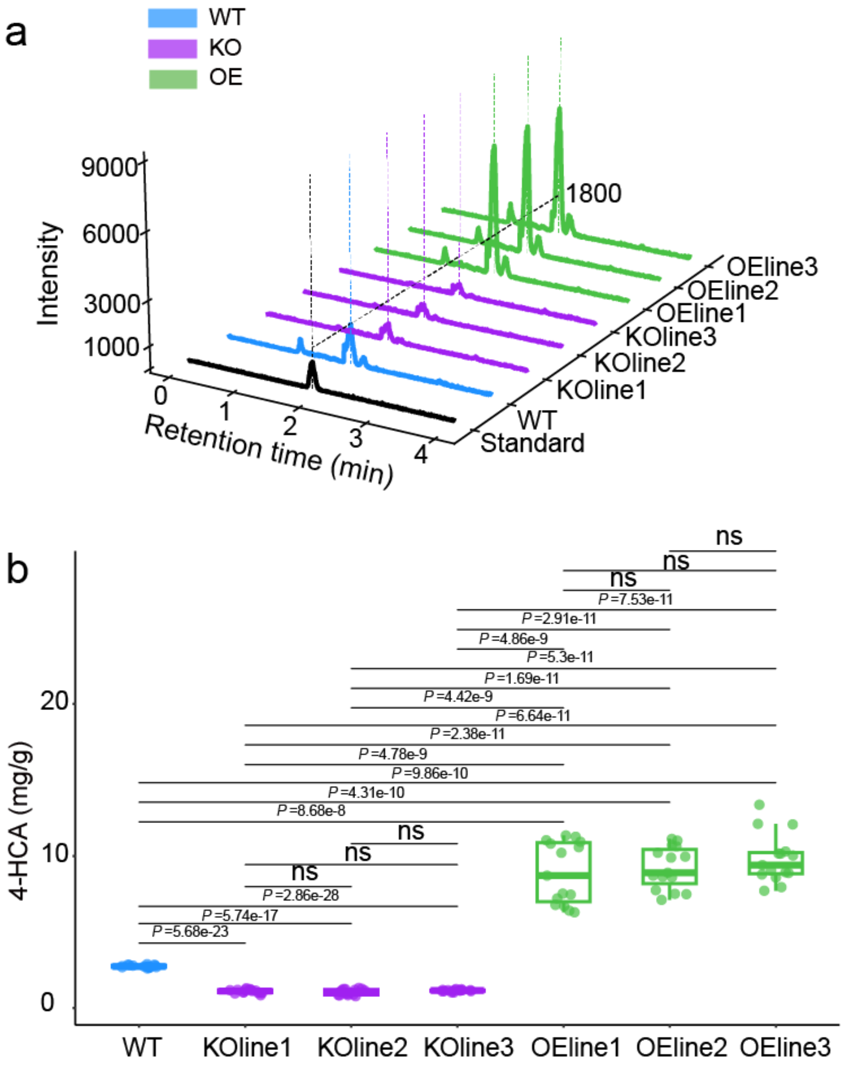

Supplementary Fig. 11. Concentration of 4-HCA in WT, KO and OE lines. (**a**) Comparative LC-MS total ion chromatograms (TICs) of leave extracts of the WT (wild-type, ZH11 rice variety), KOline1, KOline2, KOline3, OEline1, OEline2 and OEline3 plants and the 4-HCA standard. (**b**) Comparison of the concentrations of 4-HCA in WT, KO and OE lines. The numbers of replicated samples are as follows: WT (n=15), KOline1(n=15), KOline2 (n=15), KOline3 (n=15), OEline1 (n=15), OEline2 (n=15) and OEline3 (n=15). The *P*-values were calculated through unpaired one-way ANOVA with Tukey's HSD test. The labels 'ns' indicate a not significant difference ($P > 0.05$). In this figure, box plot percentiles are the same as in Supplementary Fig. 1. Blue, purple and green colors represent WT (wild-type), KO (*OsPAL02*-knockdown) and OE (*OsPAL02*-overexpression) rice plant genotypes, respectively. Groups are abbreviated as: wild-type, WT; *OsPAL02*-knockdown line 2, KOline2; *OsPAL02*-knockdown line 3, KOline3; *OsPAL02*-overexpression line 2, OEline2; *OsPAL02*-overexpression line 3, OEline3. Source data are provided as a Source Data file.

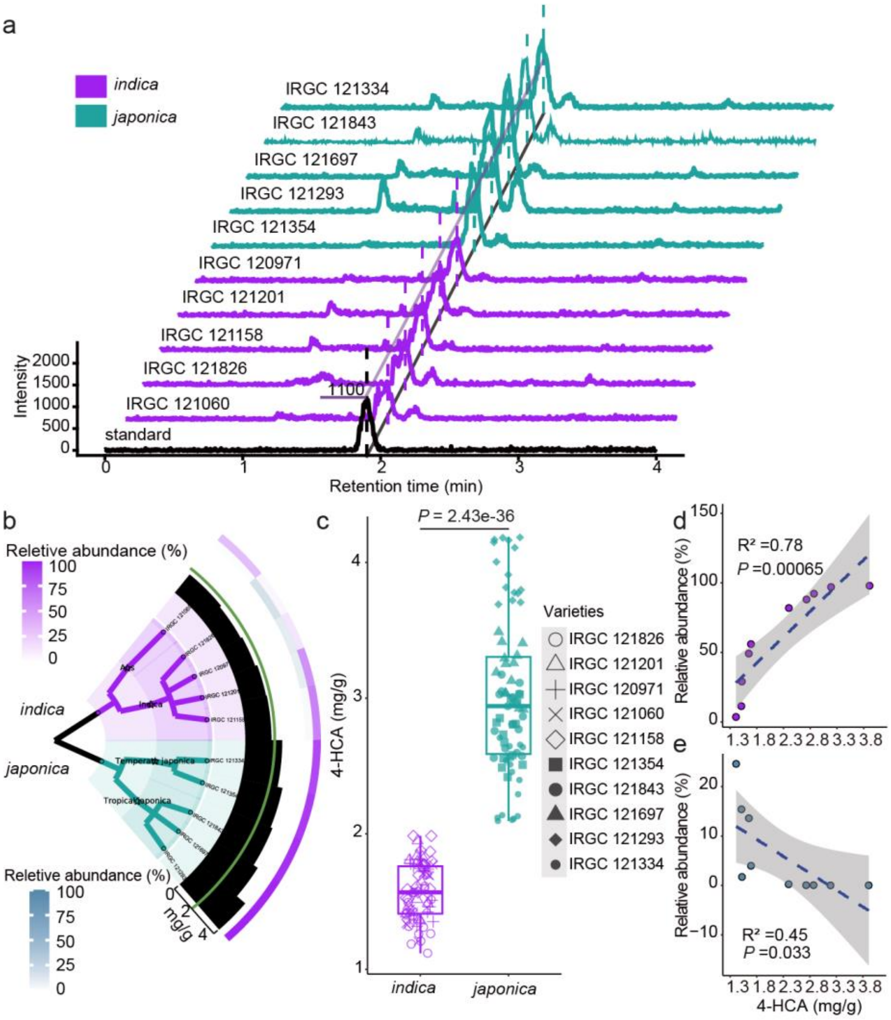

Supplementary Fig. 12. Concentration of 4-HCA in *indica* and *japonica* varieties. (**a**) Comparative LC-MS total ion chromatograms (TICs) of leaf extracts of five *indica* varieties (IRGC 121826, IRGC 121201, IRGC 120971, IRGC 121060 and IRGC 121158) and five *japonica* varieties (IRGC 121354, IRGC 121843, IRGC 121697, IRGC 121293 and IRGC 121334), and the 4-HCA standard. (**b**) Correlations between leave concentration of 4-HCA and phyllosphere microbiomes of ten rice varieties. The inner part represents the phylogeny of the rice variety. The outer purple and green rings represent relative abundances of Pseudomonadales and Xanthomonadales respectively. Black bars indicate concentrations of 4-HCA in rice varieties. (**c**) Comparison of concentration of 4-HCA in *indica* (n=5) and *japonica* (n=5) varieties. The numbers of replicated samples of each variation are 15. The *P*-values were calculated with unpaired one-way ANOVA with Tukey's HSD test. In this figure, box plot percentiles are the same as in Supplementary Fig. 1. Different shapes indicated different rice variations. Purple and green colors represent *indica* and *japonica* subgroups, respectively. (**d** & **e**) Correlation analyses between the mean of concentration of 4-HCA and the mean relative abundances of Pseudomonadales (**d**) and Xanthomonadales (**e**) in 10 rice varieties. The two-sided Spearman coefficient R^2 (*P*-value < 0.01) was calculated using ggplot2, grey areas show the 95% confidence interval of the regression line (black dashed line). Source data are provided as a Source Data file.

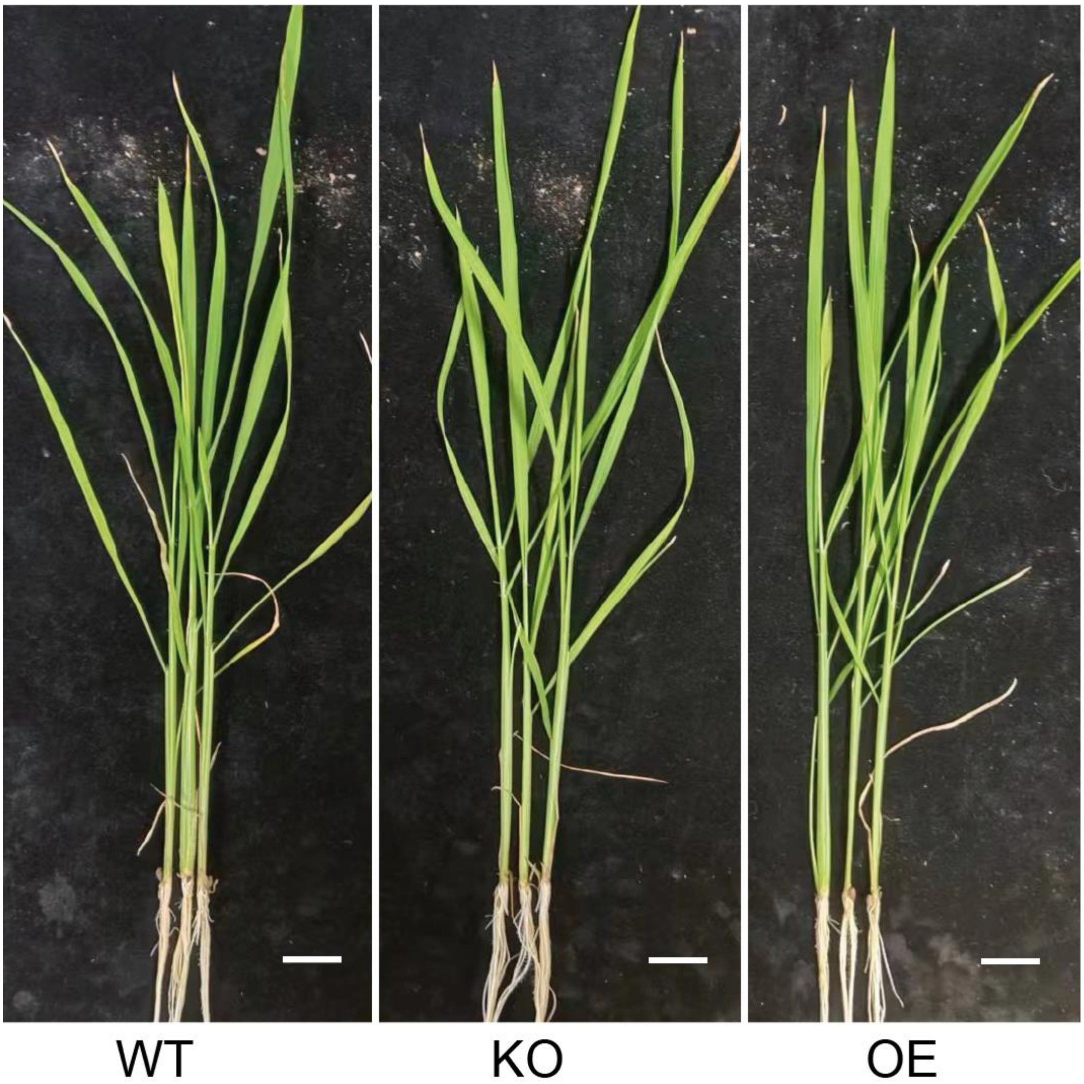

Supplementary Fig. 13. Phenotypes of WT, KO and OE rice before leaf cutting. Groups are abbreviated as: wild-type, WT; *OsPAL02*-knockdown mutant, KO; *OsPAL02*-overexpression mutant, OE. Scale bar, 1 cm.

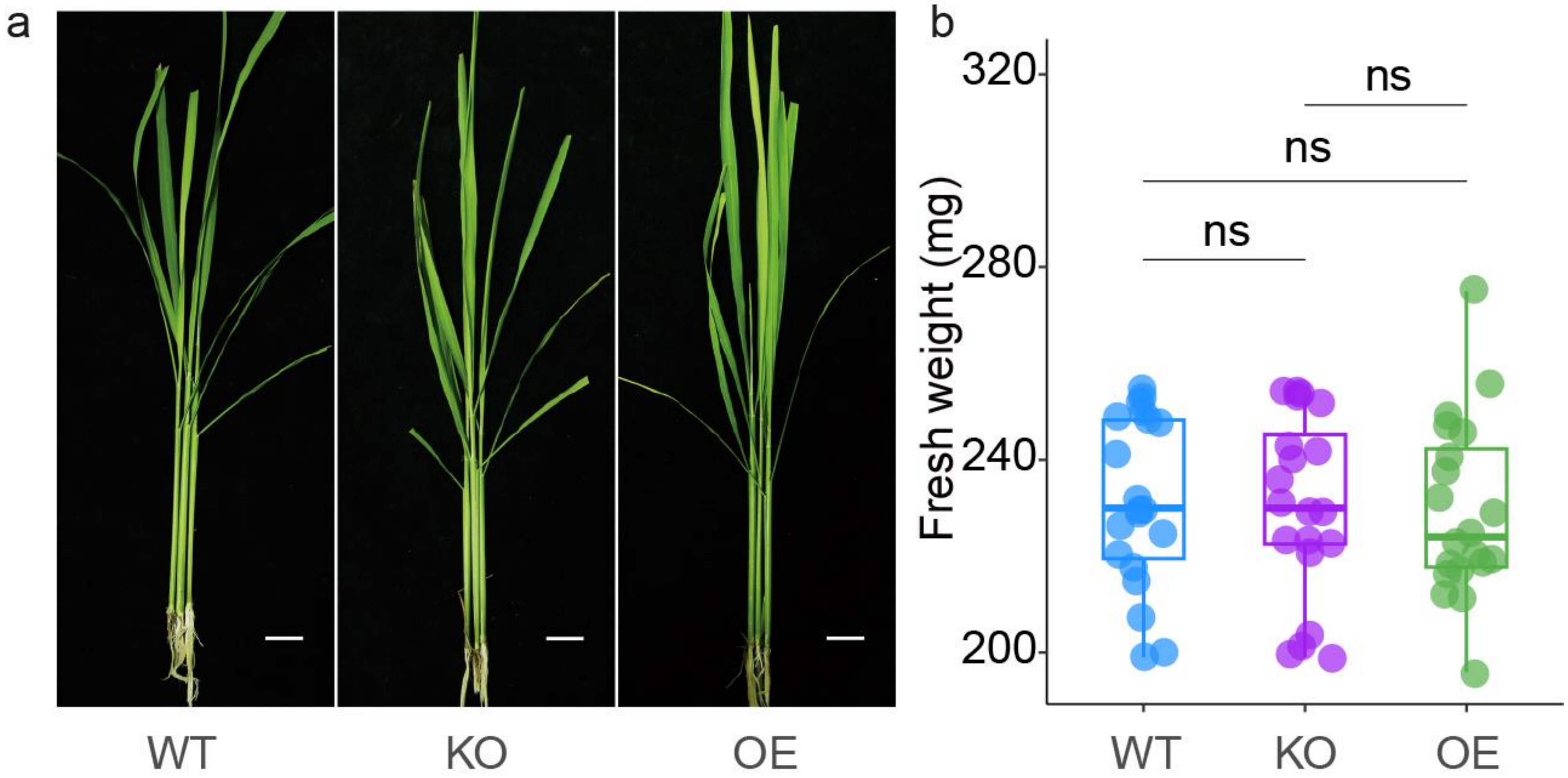

Supplementary Fig. 14. Phenotypes of WT, KO and OE rice grown under gnotobiotic conditions. Representative images of typical phenotypic traits (**a**) and fresh weight (**b**) between WT, KO and OE rice plants cultivated under gnotobiotic conditions. The numbers of replicated samples are as follows: WT (n=20), KO (n=20) and OE (n=20). Scale bar, 1 cm. The labels 'ns' indicate a not significant difference assessed with unpaired one-way ANOVA with Tukey's HSD test ($P > 0.05$). In this figure, box plot percentiles are the same as in Supplementary Fig. 1. Group are abbreviated as: wild-type, WT; *OsPAL02*-knockdown, KO; *OsPAL02*-overexpression, OE. Source data are provided as a Source Data file.

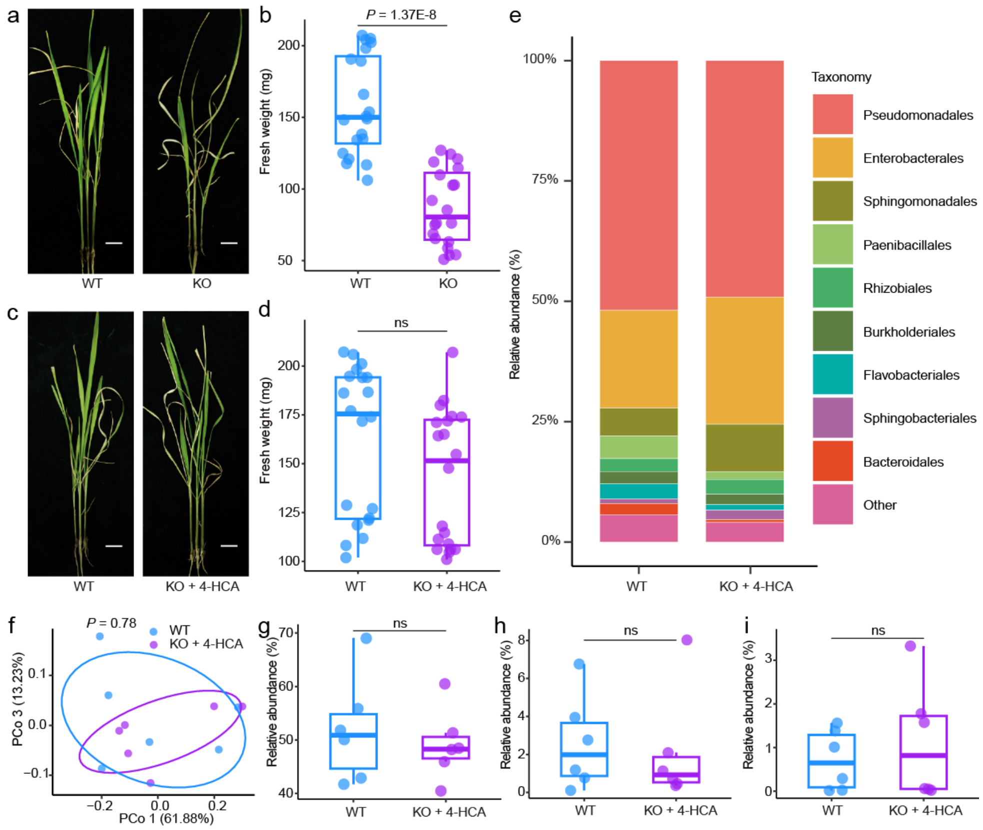

Supplementary Fig. 15. *OsPAL02*-KO plants supplemented with 4-HCA preventing microbiome dysbiosis. (**a** and **b**) Representative image of typical phenotypic traits (**a**) and fresh weight (**b**) between WT, and KO rice plants cultivated under greenhouse conditions. (**c** and **d**) Representative images of typical phenotypic traits (**c**) and fresh weight (**d**) between WT, and KO+4-HCA rice plants. The numbers of replicated samples are as follows: WT (n=20), KO (n=20) and KO+4-HCA (n=20). (**e**) Order-level composition of WT and KO phyllosphere microbiomes. (**f**) Unconstrained PCoA of Bray-Curtis distances showing the phyllosphere microbiome separation of WT and KO+4-HCA plants in the first two axes (P = 0.78, P-value was calculated with PERMANOVA). Ellipses cover 68% of the data for each group. (**g**, **h** and **i**) Statistical analysis of Pseudomonadales (**g**), Burkholderiales (**h**) and Xanthomonadales (**i**) relative abundance between WT and KO+4-HCA phyllosphere microbiomes. The numbers of replicated samples in **e**, **f**, **g**, **h** and **i** are as follows: WT(n=6) and KO+4-HCA(n=6). The P-values were calculated with unpaired one-way ANOVA with Tukey's HSD test. The labels 'ns' indicate a not significant difference (P > 0.05). Scale bar, 1 cm. In this figure, box plot percentiles are the same as in Supplementary Fig. 1. Group are abbreviated as: wild-type, WT; *OsPAL02*-knocdown, KO; *OsPAL02*-knockdown supplemented with 4-HCA, KO+4-HCA. Source data are provided as a Source Data file.

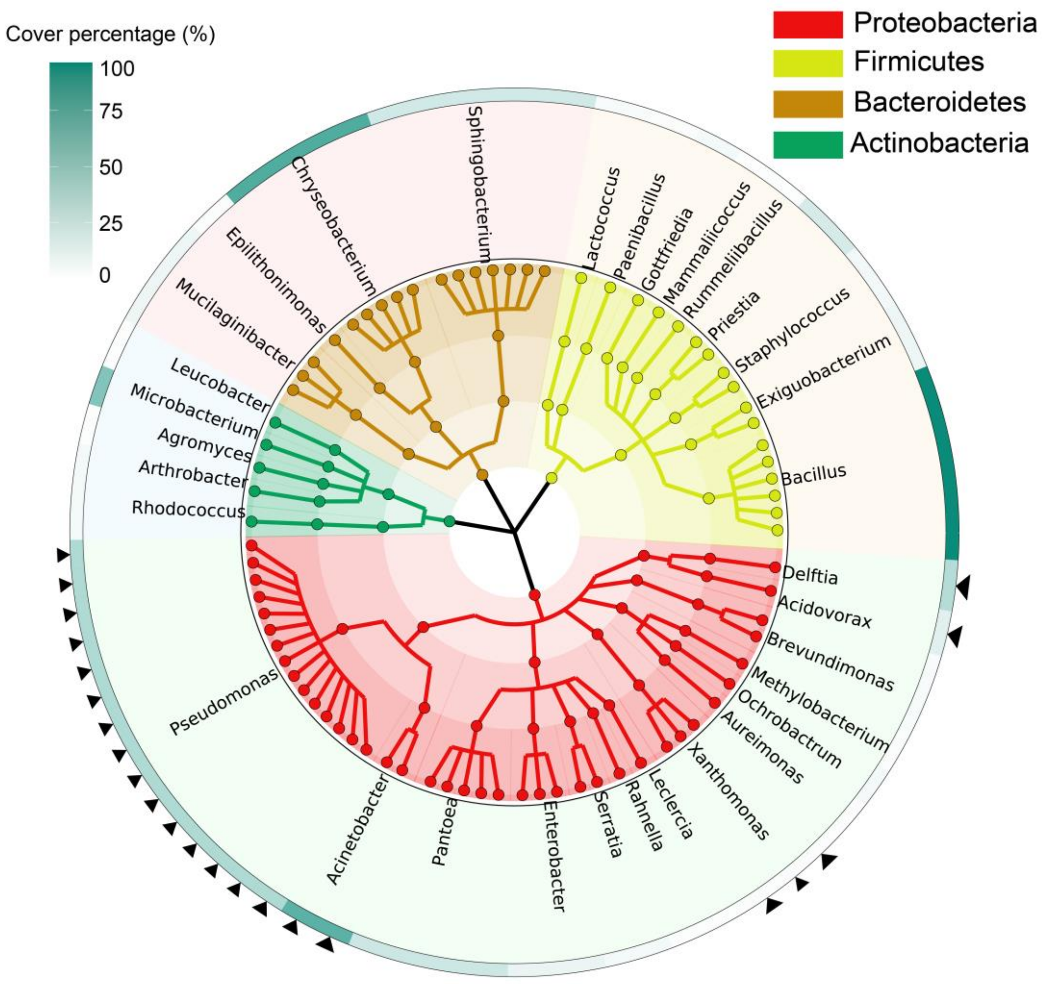

Supplementary Fig. 16. Bacterial genera in rice leaf-associated bacterial culture collection. The inner part represents the phylogeny of all species that were isolated from WT, KO and OE rice phyllospheres. The outer green ring represents the coverage percentage when compared with the respective abundance in the microbiome data. Black triangles indicate isolates that are used in this study. Source data are provided as a Source Data file.

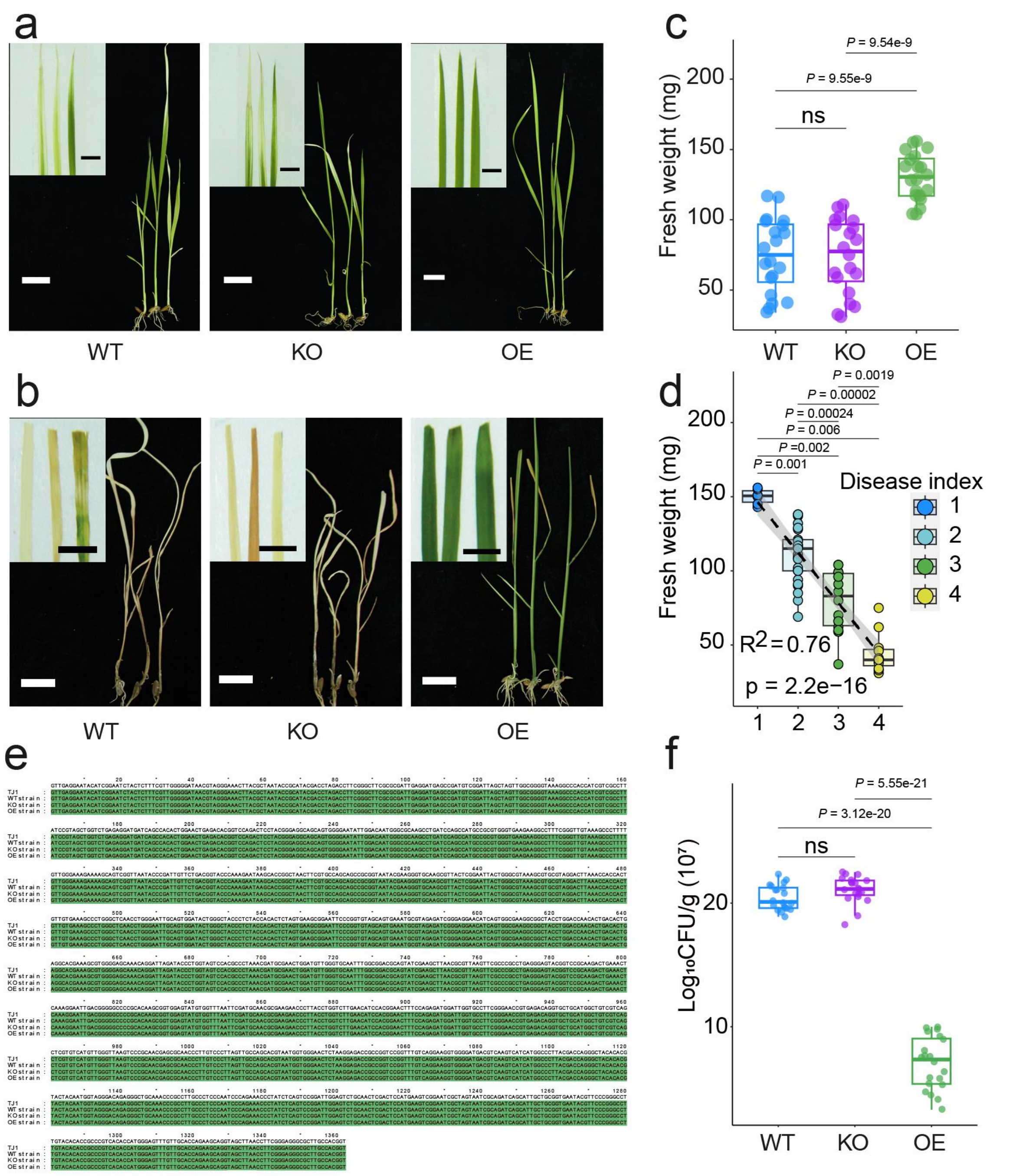

Supplementary Fig. 17. *Xanthomonas oryzae* TJ1 was identified as opportunistic pathogen causing distinct disease symptoms. (**a**) Representative images of typical phenotypic traits between WT, KO and OE plants inoculated with *Xanthomonas oryzae* TJ1 (abbreviation TJ1) before leaf cutting. (**b**) Representative images of typical phenotypic traits and fresh weight (**c**) between WT, KO and OE inoculated with TJ1 after leaf cutting. (**d**) Correlation analysis between disease index categories (1, healthy, to 4, dead) and plant fresh weight for rice plants inoculated with TJ1 after leaf cutting. The two-sided

Spearman coefficient R^2 (P-value < 0.01) was calculated using ggplot2, grey area shows the 95% confidence interval of the regression line (black dashed line). Scale bar, 5 mm. The numbers of replicated samples in each treatment are as follows: WT (n=20), KO (n=20) and OE (n=20). (**e**) Alignment of 16S rRNA gene sequences between TJ1, WT-re-isolated bacteria, KO-re-isolated bacteria and OE-re-isolated bacteria using cluster W. Green color indicate sequence similarity at 100%. (**f**) Bacterial isolation from WT, KO and OE plant leaves that were inoculated with TJ1. The numbers of replicated samples in each treatment are as follows: WT (n=20), KO (n=20) and OE (n=20). In this figure, box plot percentiles are the same as in Supplementary Fig. 1. The P-values were calculated with unpaired one-way ANOVA with Tukey's HSD test. The labels 'ns' indicate a not significant difference (P > 0.05). Groups are abbreviated as: wild-type, WT; *OsPAL02*-knockdown, KO; *OsPAL02*-overexpression, OE. Source data are provided as a Source Data file.

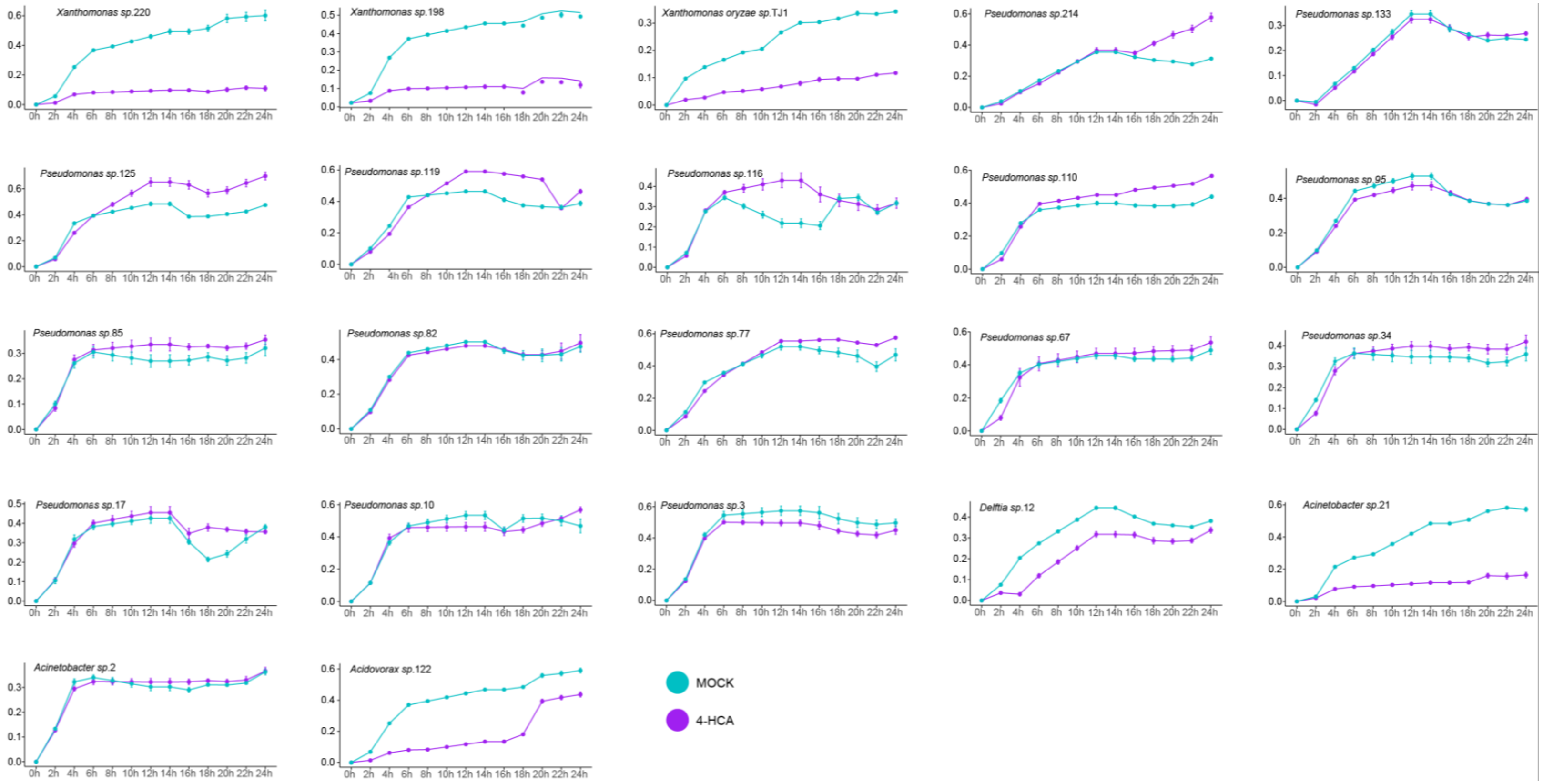

Supplementary Fig. 18. Growth curves of bacteria co-cultured with 4-HCA. 4-HCA selectively inhibits *in vitro* growth of rice phyllosphere-associated bacterial isolates. Groups are abbreviated as: R2A supplemented with 2 mM 4-HCA, 4-HCA; R2A supplemented with equivalent of solvent, MOCK. Values are means ± SD of OD_{600} (shown as error bars, n=6). Source data are provided as a Source Data file.

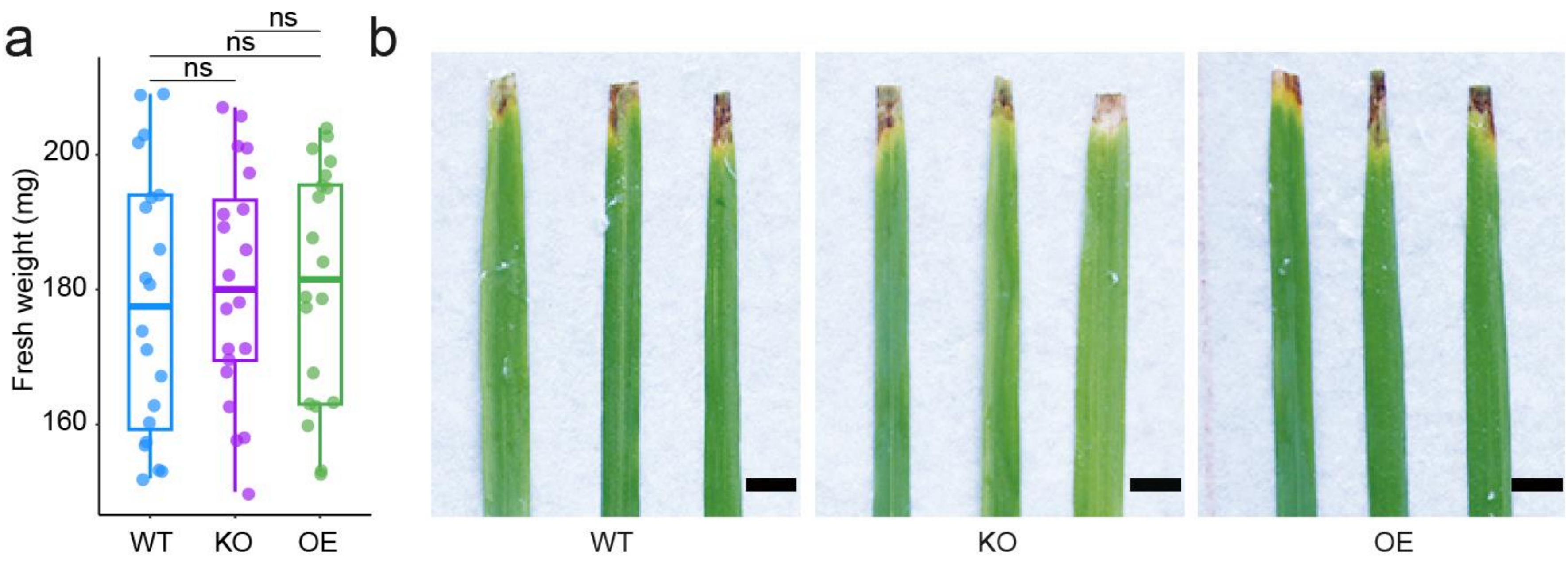

Supplementary Fig. 19. Phenotypes of WT, KO and OE rice grown inoculated with synthetic community under gnotobiotic conditions. (**a** and **b**) Representative images of typical phenotypic traits (**b**) and fresh weight (**a**) between gnotobiotically cultivated WT, KO and OE rice plants inoculated with synthetic community (abbreviated as SynCom). In this figure, box plot percentiles are the same as in Supplementary Fig.1. Blue, purple and green colors represent WT, KO and OE rice plants, respectively. The labels ‘ns’ indicate a not significant difference assessed with unpaired one-way ANOVA with Tukey’s HSD test ($P > 0.05$). The numbers of replicated samples in each treatment are as follows: WT (n=20), KO (n=20) and OE (n=20). Scale bar, 5 mm. Groups are abbreviated as: wild-type, WT; *OsPAL02*-knocdown, KO; *OsPAL02*-overexpression, OE. Source data are provided as a Source Data file.

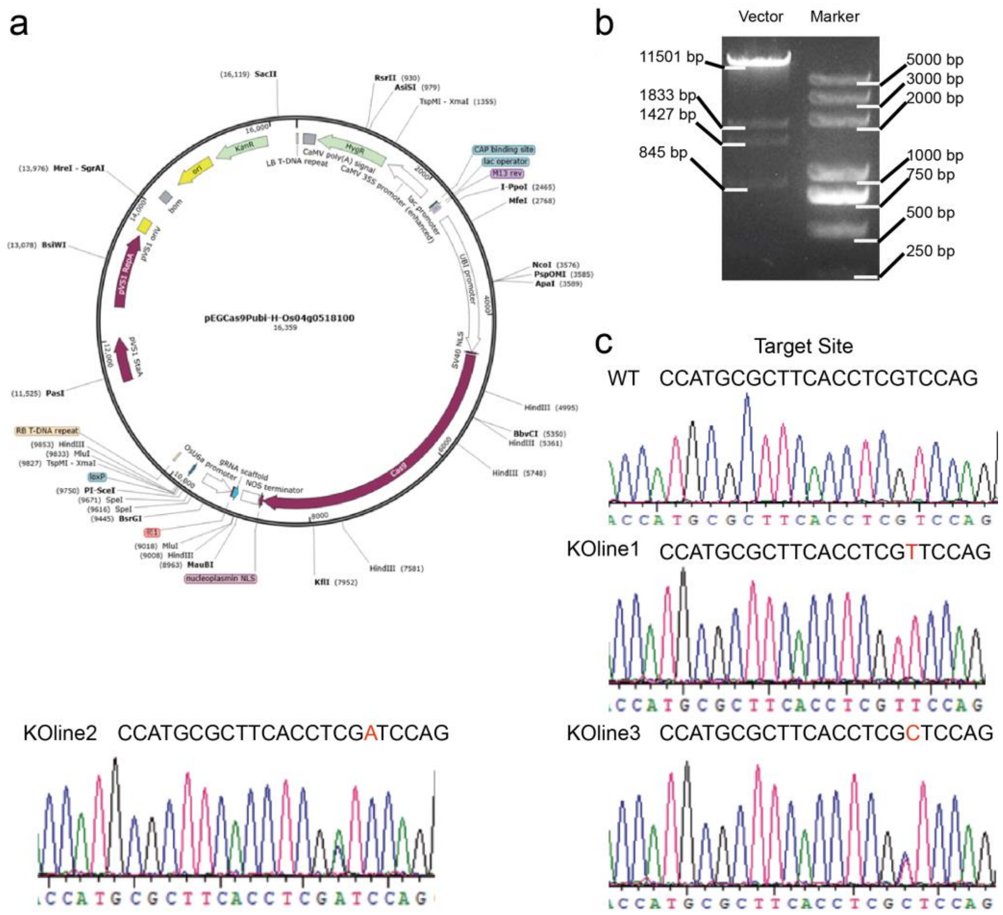

Supplementary Fig. 20. Generation of *OsPAL02* (Os04g0518100) KO lines. (**a**) The vector pEGCas9Pubi-H-Os04g0518100 was used to generate CRISPR–Cas9 edited rice plants. (**b**) pEGCas9Pubi-H-Os04g0518100 was enzyme-digested with HindIII. (**c**) Sequences of WT and KO lines at CRISPR–Cas9 target site. Source data are provided as a Source Data file.

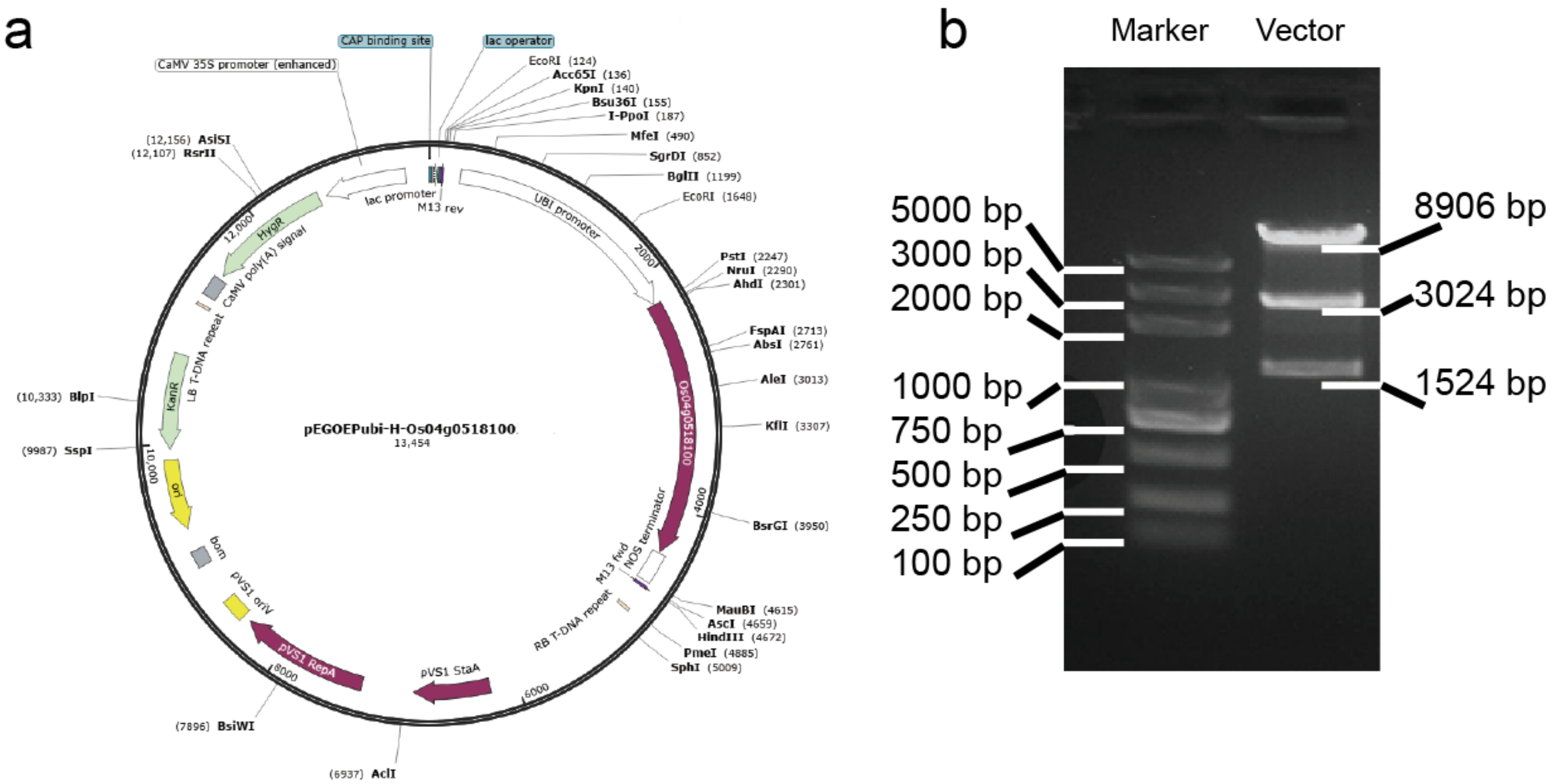

Supplementary Fig. 21. Generation of *OsPAL02* (Os04g0518100) OE lines. (**a**) The vector pEGOEPubi-H-Os04g0518100 was used to generate *OsPAL02*-overexpression rice plants. (**b**) pEGOEPubi-H-Os04g0518100 was enzyme-digested with EcoRI/HindIII. Source data are provided as a Source Data file.

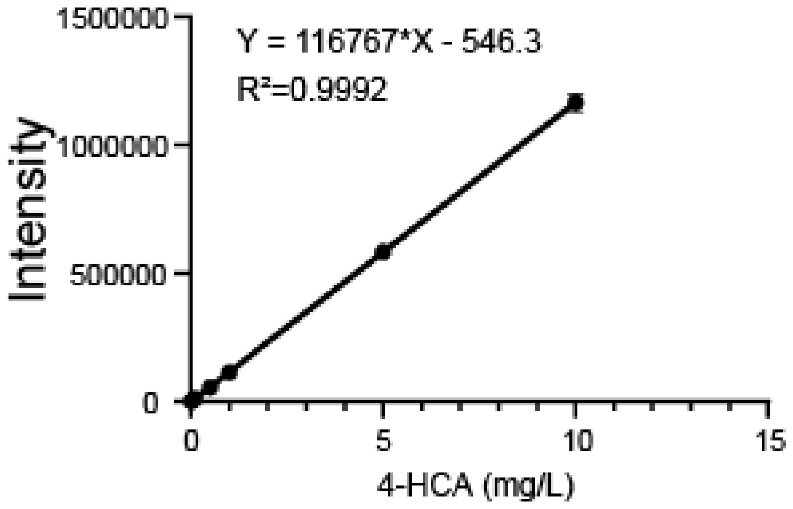

Supplementary Fig. 22. Standard curve for 4-HCA quantification. A standard curve was generated to assess the relationship between 4-HCA concentration and LC-MS ion intensity using linear regression. Source data are provided as a Source Data file.
