# Supplementary material for: Microbiome homeostasis on rice leaves is regulated by a precursor molecule of lignin biosynthesis
Source: Nat Commun. 2024 Jan 2;15:23. doi: 10.1038/s41467-023-44335-3 (PMC10762202; doi:10.1038/s41467-023-44335-3)
Supplement: Supplementary file 3 — Description of Additional Supplementary Files [file 41467_2023_44335_MOESM3_ESM.pdf]

Description of Additional Supplementary Files

File Name: Supplementary Data 1
Description: Information on rice varieties used for metagenome sequencing.

File Name: Supplementary Data 2
Description: Microbiome profiles of phyllospheres from 110 rice varieties.

File Name: Supplementary Data 3
Description: GWAS analysis with phyllosphere microbiomes.

File Name: Supplementary Data 4
Description: GWAS locus clustering and metabolism pathway analysis.

File Name: Supplementary Data 5
Description: P-values for unpaired one-way ANOVA with Tukey's HSD test for 16S rRNA gene amplicon analysis and metabolite analysis.

File Name: Supplementary Data 6
Description: Metabolite analysis of *OsPAL02*- WT, KO and OE rice leaves.

File Name: Supplementary Data 7
Description: Bacterial isolation and co-cultivation with 4-HCA.

File Name: Supplementary Data 8
Description: SNPs of 110 rice varieties.
