# Supplementary material for: Microbiome homeostasis on rice leaves is regulated by a precursor molecule of lignin biosynthesis
Source: Nat Commun. 2024 Jan 2;15:23. doi: 10.1038/s41467-023-44335-3 (PMC10762202; doi:10.1038/s41467-023-44335-3)
Supplement: Supplementary file 12 — Reporting Summary [file 41467_2023_44335_MOESM12_ESM.pdf]

nature portfolio

Corresponding author(s): Deyon Zhang, Tomislav Cernava and Yong Liu

Last updated by author(s): 14/11/23

Reporting Summary

Nature Portfolio wishes to improve the reproducibility of the work that we publish. This form provides structure for consistency and transparency in reporting. For further information on Nature Portfolio policies, see our Editorial Policies and the Editorial Policy Checklist.

Statistics

For all statistical analyses, confirm that the following items are present in the figure legend, table legend, main text, or Methods section.

n/a	Confirmed	
☐	☒	The exact sample size (*n*) for each experimental group/condition, given as a discrete number and unit of measurement
☐	☒	A statement on whether measurements were taken from distinct samples or whether the same sample was measured repeatedly
☐	☒	The statistical test(s) used AND whether they are one- or two-sided<br>*Only common tests should be described solely by name; describe more complex techniques in the Methods section.*
☒	☐	A description of all covariates tested
☐	☒	A description of any assumptions or corrections, such as tests of normality and adjustment for multiple comparisons
☐	☒	A full description of the statistical parameters including central tendency (e.g. means) or other basic estimates (e.g. regression coefficient) AND variation (e.g. standard deviation) or associated estimates of uncertainty (e.g. confidence intervals)
☐	☒	For null hypothesis testing, the test statistic (e.g. *F*, *t*, *r*) with confidence intervals, effect sizes, degrees of freedom and *P* value noted<br>*Give P values as exact values whenever suitable.*
☒	☐	For Bayesian analysis, information on the choice of priors and Markov chain Monte Carlo settings
☒	☐	For hierarchical and complex designs, identification of the appropriate level for tests and full reporting of outcomes
☒	☐	Estimates of effect sizes (e.g. Cohen's *d*, Pearson's *r*), indicating how they were calculated

Our web collection on statistics for biologists contains articles on many of the points above.

Software and code

Policy information about availability of computer code

Data collection	Data collection was done with commercial and open source software available at the institutions of the authors.<br>LC-MS/MS data was collected using AB SCIEX Analyst TF v1.6 (cinnamic acid and derivatives profiling) and AB SCIEX Analyst v1.6 (quantification of 4-HCA in rice leaves). Microbiome sequencing was performed on the Illumina NovaSeq 6000 platform.
Data analysis	Data analysis was performed with different commercial and open source software as follows:<br>Microbiome data analysis was conducted using Trimmomatic v0.39, VSEARCH v2.14.2, Kraken2 v2.0.9, Bracken v2.6.0, GraPhlAn v.0.9.7, QIIME2 v.2021.4 , Cutadapt v.2.1, DADA2 v.2020.2.0, Silva v138.1 and R package ggplot2 v.2.2.1, vegan in R 4.1.0.<br>GWAS and data analysis were conducted using TASSEL 5.0, STRUCTURE 2.3.4, Activeperl v5.8 and Java environment openjdk version "1.8.0_161" with Linux system version 3.10.0-862.el7.x86_64 and R package circlize and clusterProfile in R 4.1.0.<br>Metabolite data analyses were performed using ProteoWizard MSConvert and R package XCMS v3.20.0, CAMERA v3.1-5, ComplexHeatmap and ggplot2 v.2.2.1 in R 4.1.0.<br>Scripts employed in the computational analyses are available at https://github.com/kanghouxiang105/micro-GWAS and https://zenodo.org/records/10115039.

For manuscripts utilizing custom algorithms or software that are central to the research but not yet described in published literature, software must be made available to editors and reviewers. We strongly encourage code deposition in a community repository (e.g. GitHub). See the Nature Portfolio guidelines for submitting code & software for further information.

Data

Policy information about availability of data

All manuscripts must include a data availability statement. This statement should provide the following information, where applicable:

- Accession codes, unique identifiers, or web links for publicly available datasets
- A description of any restrictions on data availability
- For clinical datasets or third party data, please ensure that the statement adheres to our policy

Raw sequence data (16S rRNA gene fragment sequencing) reported in this work were deposited in the Genome Sequence Archive of the BIG Data Center(77), Chinese Academy of Sciences under the project PRJCA016320 publicly accessible at http://bigd.big.ac.cn/gsa. The metagenomes of the phyllosphere of 110 rice genotypes were deposited in the European Nucleotide Archive (ENA) database under the project PRJEB45634 (https://www.ebi.ac.uk/ena/browser/view/PRJEB45634). The 4-HCA-associated pathway is available under the pathway map “dosa00940” in the KEGG Pathway database (https://www.kegg.jp). MiniKraken2_v1_8GB database downloaded from the genome website browser (http://ccb.jhu.edu/software/bracken/) was used for taxonomic classification of shotgun-microbiome. Silva v138.1 reference databases downloaded from the website browser (https://www.arb-silva.de/documentation/release-1381/) was used for taxonomic classification of 16S rRNA-microbiome. Rice genes annotated at Rice Annotation Project Database (RAPDB, https://rapdb.dna.affrc.go.jp/). GO function annotated at org.Osativa.eg.db (https://github.com/xuzhougeng/org.Osativa.eg.db) database. All isolated bacterial strains and transformed rice lines were deposited in the State Key Laboratory of Hybrid Rice and the Institute of Plant Protection of Hunan Academy of Agricultural Sciences (Changsha, China). Other data generated in this study are available in the Supplementary Data files. Source data are provided with this paper.

Research involving human participants, their data, or biological material

Policy information about studies with human participants or human data. See also policy information about sex, gender (identity/presentation), and sexual orientation and race, ethnicity and racism.

Reporting on sex and gender	not applicable
Reporting on race, ethnicity, or other socially relevant groupings	not applicable
Population characteristics	not applicable
Recruitment	not applicable
Ethics oversight	not applicable

Note that full information on the approval of the study protocol must also be provided in the manuscript.

Field-specific reporting

Please select the one below that is the best fit for your research. If you are not sure, read the appropriate sections before making your selection.

☒ Life sciences ☐ Behavioural & social sciences ☐ Ecological, evolutionary & environmental sciences

For a reference copy of the document with all sections, see nature.com/documents/nr-reporting-summary-flat.pdf

Life sciences study design

All studies must disclose on these points even when the disclosure is negative.

Sample size	No statistical methods were used to predetermine sample sizes. The sample size and the results of statistical analyses are described in the relevant figure legends. Sample size was determined based on requirements for statistical analysis, attempting to have a minimum of six biological replicates with sufficient reproducibility.
Data exclusions	No data points were excluded from analysis in any experiment described in this manuscript.
Replication	The number of replication for each experiment (at least three repeats) is described in the relevant figure legends.
Randomization	Groups were allocated based on the genetic background of plants, thus no randomization was required. Seeds of the same genotype were randomly allocated to experimental groups. To avoid edge effects (variations in light, temperature and humidity) on plant growth, plants were randomly placed in the growth chamber and their position was changed every 2-3 days.
Blinding	Sample collection was done blindly as the plants were marked by a code rather than by plant genotype or treatment.

Reporting for specific materials, systems and methods

We require information from authors about some types of materials, experimental systems and methods used in many studies. Here, indicate whether each material, system or method listed is relevant to your study. If you are not sure if a list item applies to your research, read the appropriate section before selecting a response.

Materials & experimental systems

n/a	Involved in the study
☒	☐ Antibodies
☒	☐ Eukaryotic cell lines
☒	☐ Palaeontology and archaeology
☒	☐ Animals and other organisms
☒	☐ Clinical data
☒	☐ Dual use research of concern
☐	☒ Plants

Methods

n/a	Involved in the study
☒	☐ ChIP-seq
☒	☐ Flow cytometry
☒	☐ MRI-based neuroimaging

Dual use research of concern

Policy information about dual use research of concern

Hazards

Could the accidental, deliberate or reckless misuse of agents or technologies generated in the work, or the application of information presented in the manuscript, pose a threat to:

No	Yes
☒	☐ Public health
☒	☐ National security
☒	☐ Crops and/or livestock
☒	☐ Ecosystems
☒	☐ Any other significant area

Experiments of concern

Does the work involve any of these experiments of concern:

No	Yes
☒	☐ Demonstrate how to render a vaccine ineffective
☒	☐ Confer resistance to therapeutically useful antibiotics or antiviral agents
☒	☐ Enhance the virulence of a pathogen or render a nonpathogen virulent
☒	☐ Increase transmissibility of a pathogen
☒	☐ Alter the host range of a pathogen
☒	☐ Enable evasion of diagnostic/detection modalities
☒	☐ Enable the weaponization of a biological agent or toxin
☒	☐ Any other potentially harmful combination of experiments and agents
